# Bcl-xL in neuroprotection and plasticity

**DOI:** 10.3389/fphys.2014.00355

**Published:** 2014-09-17

**Authors:** Elizabeth A. Jonas, George A. Porter, Kambiz N. Alavian

**Affiliations:** ^1^Section of Endocrinology, Department of Internal Medicine, Yale UniversityNew Haven, CT, USA; ^2^Department of Neurobiology, Yale UniversityNew Haven, CT, USA; ^3^Departments of Pediatrics (Cardiology), University of Rochester Medical CenterRochester, NY, USA; ^4^Internal Medicine (Aab Cardiovascular Research Institute), University of Rochester Medical CenterRochester, NY, USA; ^5^Department of Pharmacology and Physiology, University of Rochester Medical CenterRochester, NY, USA; ^6^Division of Brain Sciences, Department of Medicine, Imperial College LondonLondon, UK

**Keywords:** apoptosis, mitochondria, synaptic plasticity, calcium, permeability transition pore

## Abstract

Accepted features of neurodegenerative disease include mitochondrial and protein folding dysfunction and activation of pro-death factors. Neurons that experience high metabolic demand or those found in organisms with genetic mutations in proteins that control cell stress may be more susceptible to aging and neurodegenerative disease. In neurons, events that normally promote growth, synapse formation, and plasticity are also often deployed to control neurotoxicity. Such protective strategies are coordinated by master stress-fighting proteins. One such specialized protein is the anti-cell death Bcl-2 family member Bcl-xL, whose myriad death-protecting functions include enhancement of bioenergetic efficiency, prevention of mitochondrial permeability transition channel activity, protection from mitochondrial outer membrane permeabilization (MOMP) to pro-apoptotic factors, and improvement in the rate of vesicular trafficking. Synapse formation and normal neuronal activity provide protection from neuronal death. Therefore, Bcl-xL brings about synapse formation as a neuroprotective strategy. In this review we will consider how this multi-functional master regulator protein uses many strategies to enhance synaptic and neuronal function and thus counteracts neurodegenerative stimuli.

## Introduction

**Figure 1:**
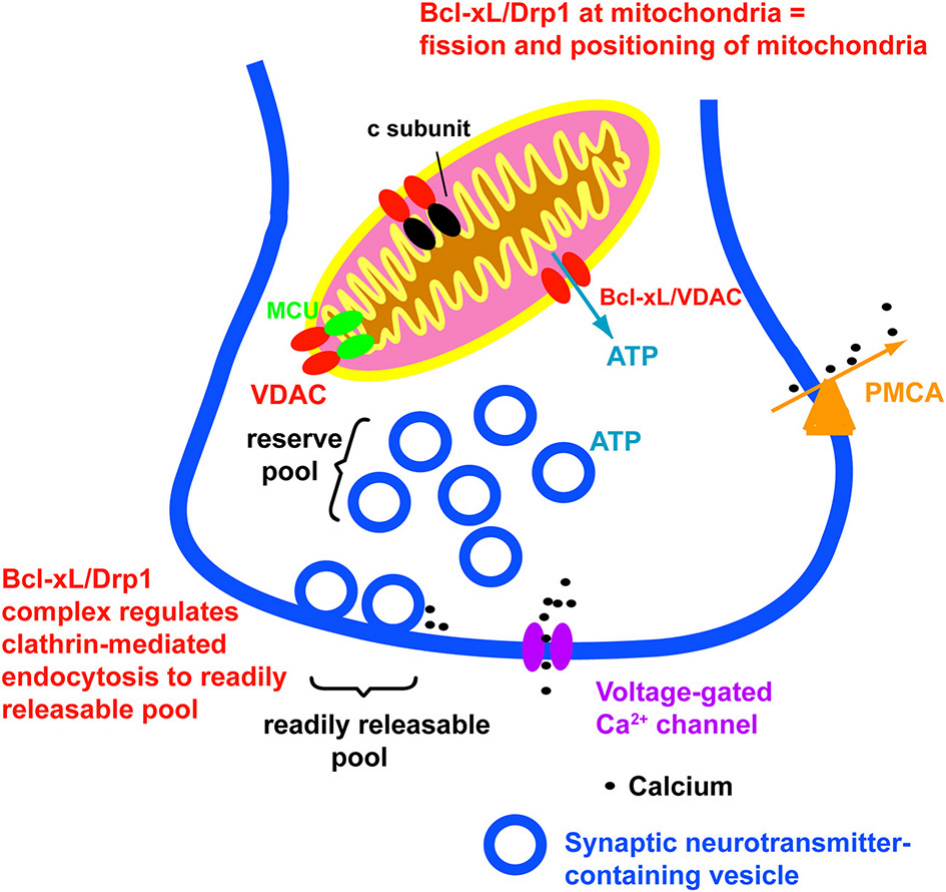
**Bcl-xL affects aspects of normal neuronal function and plasticity**. These include release of ATP from mitochondria, closing of a leak conductance in the mitochondrial inner membrane, modulation of mPTP, and regulation of synaptic vesicle recycling. Not illustrated is the canonical role of Bcl-xL in prevention of activation of pro-apoptotic factors.

Recent discoveries have shed light on the increasingly complex and varied activities of Bcl-2 family proteins. Discovery of novel roles for these proteins in the nervous system suggests that protection from cell death is a complex operation that is integrated with other important cellular pathways. The anti-apoptotic protein Bcl-xL has been found to regulate mitochondrial bioenergetic efficiency, synaptic transmitter release, synaptic vesicle recycling and neurite growth (Figure 1) in addition to its canonical role in preventing the activity of pro-apoptotic proteins. Although it is tempting to assign a primary function, such as regulation of metabolism or cell death (survival), none of these roles appears obviously more important than any other. In the nervous system, proteins that have evolved to regulate differentiation, synaptic connectivity and survival during development are used in adult life to increase cellular efficiency and to enhance synaptic plasticity. Bcl-xL expression is maintained throughout life, perhaps to make new synaptic connections and to potentiate neuronal connections. These features may help in neurite or synapse competition between stronger and weaker members, promoting neuronal soma and process “survival.” If the supposition is true, then perhaps Bcl-xL is one of a number of proteins that plays a central role not only in cellular differentiation but also in functional adaptation during adult life in order to maintain cell usefulness to prevent degeneration.

Following that line of reasoning, one can posit that neurodegenerative disease is related to at least two phenomena: Disruption of the roles of protective multifunctional proteins or passive neglect. For example, neuronal overuse over a lifetime may compromise the metabolic and protein milieu, causing a vicious cycle of damage and increased inefficiency. In contrast, if synaptic connections become inactive over time, those synapses may undergo structural and metabolic remodeling leading to decreased metabolic and neuronal network efficiency. Overuse and underuse may both, therefore, result in degeneration, especially in inherently vulnerable neuronal populations.

In this review, we will look at the role of Bcl-xL from the point of view of its multiple closely connected roles in metabolism, growth, synaptic connectivity and plasticity. We will reflect on how increases in metabolic efficiency may underlie effective synaptic and growth enhancement. We will then raise the possibility that a decrease in activity may be correlated with long term depression of neuronal synaptic and electrical activities followed by synapse deconstruction, axonal retraction and eventual somatic loss.

## Bcl-xL regulates mitochondria during neurotransmitter release

Many of the features of synaptic transmission can be enhanced over the short and long term (Bliss and Collingridge, 1993). These include changes in presynaptic calcium levels, changes in vesicle numbers and probability of release, and alterations in postsynaptic receptor numbers and function. Such phenomena are regulated by mitochondria which provide energy in the form of ATP and buffer calcium at active synapses. Mitochondria, therefore, are required for normal synaptic transmission at high frequencies (David and Barrett, 2003; Ivannikov et al., 2013). In addition, calcium uptake and re-release by mitochondria during neurotransmission regulate short term plasticity (Blaustein et al., 1978; Friel and Tsien, 1994; Tang and Zucker, 1997; Mochida, 2011; Lee et al., 2012; Wan et al., 2012) and calcium enhances enzymatic activity of several TCA cycle enzymes (Wan et al., 1989).

Mitochondrial bioenergetics may be altered acutely in synapses that have undergone conditioning, providing for enhanced oxidative competence (Nguyen et al., 1997). Unexpectedly, the Bcl-2 family proteins that are known to regulate apoptosis through their actions at mitochondrial membranes have also been newly identified as regulators of synaptic activity. Thus, the actions of Bcl-xL—an anti-cell death Bcl-2 family member—at mitochondria, position it to influence learning, memory, and alterations in behavior (Jonas, 2006). The ways in which Bcl-xL enhances the bioenergetic functioning of a neuron and the relationship of this to alterations in other cellular and synaptic activities will be addressed.

## Neuronal activity influences mitochondrial calcium uptake and the strength of subsequent synaptic events

One key feature of synaptic transmission is marked calcium influx through glutamate receptors and voltage gated calcium channels (Fioravante and Regehr, 2011; Morris, 2013; Simms and Zamponi, 2014). After calcium entry, calcium clearance from the cytosol occurs through the actions of calcium ATPases at the plasma membrane and by buffering through uptake by intracellular stores including ER and mitochondria (Rizzuto et al., 2012; Lopreiato et al., 2014); these processes reset the normal low calcium levels present in resting synapses. The calcium that is buffered by intracellular stores is eventually re-released, providing for residual calcium in synaptic endings. Residual calcium may be necessary for certain forms of synaptic plasticity (Neher and Sakaba, 2008; Mochida, 2011). Mitochondria regulate cytosolic levels of calcium and the release of metabolites through an intricate system involving several ion channels. The machinery that takes up calcium is very important to energy production, neuronal excitability and synaptic plasticity. The discovery of the molecular substrate for the calcium uniporter ion channel (MCU) at the mitochondrial inner membrane has generated increasing interest in mechanisms of calcium management within the neuronal cell body or synapse (Kirichok et al., 2004; Baughman et al., 2011; De Stefani et al., 2011; Mallilankaraman et al., 2012b). Additional isoforms of MCU and its helper MICU that confer tissue specificity and other behaviors have now been identified (Mallilankaraman et al., 2012a; Raffaello et al., 2013). Although not completely understood yet, new findings portend that sites of calcium adjustment will determine activity-dependent energy responses of mitochondria (De Stefani and Rizzuto, 2014) that could be extremely relevant for synaptic plasticity and repair.

## Calcium influx into active neurons alters ATP synthesis and release by mitochondria

Mitochondria function symbiotically with eukaryotic cells to provide energy in the form of ATP. They take up substrates from the cytosol in the form of products of glycolysis, lipid and protein metabolism. One of the main products of glycolysis, pyruvate, is acted on by pyruvate dehydrogenase to form acetyl co-enzyme A which enters the TCA cycle. Turns of the TCA cycle synthesize NADH and FADH2 that donate their electrons to the electron transport chain. The energy of the bonds of NADH and FADH2 is used to pump H+ ions out of the matrix, creating a proton motive force that in turn drives the ATP synthase. Upon kinetic repositioning of the ATP synthase rotor, ATP is synthesized from ADP and Pi (Watt et al., 2010). The machinery required for ADP influx into the matrix including the outer membrane ion channel VDAC and the adenine nucleotide transporter (ANT) in the inner membrane are intimately linked to that of the ATP synthase (Chen et al., 2004).

Calcium regulates TCA cycle enzymes (Denton, 2009) and enzymes in the electron transport chain (Gellerich et al., 2010) to speed the process of ATP synthesis. As cytosolic calcium increases during neuronal activity, mitochondrial TCA cycle enzymatic activity is enhanced, increasing the levels of mitochondrial NADH and ATP. Calcium influx into mitochondria enhances the proportion of pyruvate dehydrogenase complex in its active, dephosphorylated form. Calcium also increases the ability of isocitrate dehydrogenase and oxoglutarate dehydrogenase to bind their substrates. The result is regulation of mitochondrial TCA enzymes by cytosolic calcium or “external regulation” as opposed to “internal regulation” that is caused by changes in NAD/NADH ratio and the ratio of ADP/ATP in the mitochondrial matrix. In neurons, increases in cytosolic calcium arise from depolarization of the plasma membrane with resultant increase in permeability to calcium through glutamate receptors and voltage gated calcium channels. In addition, mitochondria receive calcium from the ER which partners with mitochondria to respond to cytosolic calcium loads. Elevated cytosolic calcium enhances the release of calcium from IP3 receptors directly into mitochondria within a tightly coupled space between the two organelles (Rizzuto et al., 2012). Oscillatory calcium release from ER provided by IP3 receptors causes influx of calcium into the mitochondria and drives TCA cycle enzymes even more effectively than sustained elevations of mitochondrial calcium (Hajnoczky et al., 2000), most likely because sustained calcium allows re-equilibration of the signal in the matrix.

The interaction between neuronal activity, calcium influx into mitochondria and energy generation was clarified recently in a study in Drosophila neuromuscular junction (Chouhan et al., 2012). Using a complex set of imaging techniques including genetically encoded calcium/pH indicators, it was shown that neuronal activity enhanced calcium uptake by mitochondria, increased mitochondrial NAD(P)H levels and hyperpolarized the mitochondrial inner membrane potential. These events were inhibited by pharmacological agents that blocked mitochondrial calcium uptake. Interestingly, the level of cytosolic calcium remained similar in different neurons despite their very different firing rates, suggesting that a certain level of cytosolic calcium is optimum for energy production during activity. This specific cytosolic calcium level is most likely achieved by an ideal combination of calcium buffering inside, and extrusion from, the nerve ending. An exciting implication of these novel findings is that in different cell types, changes in cytosolic calcium levels could theoretically produce plasticity in mitochondrial responses to adjust to changes in energy demands in synapses undergoing long term changes in strength.

Another type of mitochondrial management of cytosolic calcium was observed in the large mammalian brain stem presynaptic terminals of the Calyx of Held. In this study, mitochondria were found to remove calcium during rises in cytosolic calcium produced by calcium influx though voltage gated channels. Mitochondrial calcium uptake occurred early in the stimulation paradigm, and dampened the overall rise in calcium in the presynaptic terminal that occurred in the absence of functional mitochondria. The effect of this was to limit neurotransmission produced by a short train of stimuli. The authors concluded that mitochondrial calcium buffering served to prevent synaptic depression by attenuating vesicle depletion and allowing for continued effective synaptic transmission during a train of action potentials (Billups and Forsythe, 2002).

In addition to calcium buffering, acute changes in mitochondrial membrane activity were found to be necessary for short term synaptic potentiation in squid presynaptic terminal. Through the use of a double-barreled patch pipette (Jonas et al., 1997) recordings were made inside the presynaptic terminal on mitochondria both at rest and during and after intense synaptic stimulation (Jonas et al., 1999). In control recordings within the resting presynaptic terminal, the conductance of mitochondrial membranes was found to be low. In contrast, during high frequency electrical stimulation of the presynaptic nerve, a large increase in mitochondrial membrane activity occurred (Jonas et al., 1999). The delay in onset of the mitochondrial activity and the persistence of the mitochondrial activity after stimulation implied that mitochondrial channel activity was not simultaneous with plasma membrane channel activity. This suggested that the increase in activity depended on an intracellular second messenger, most likely calcium (Csordas et al., 2012). In keeping with this, mitochondrial activity was abrogated by removing calcium from the bathing medium during stimulation, demonstrating that the evoked mitochondrial membrane channel activity was dependent on calcium influx into the terminal and into mitochondria (Jonas et al., 1999). The uncoupler FCCP (carbonyl cyanide *p*-trifluoromethoxyphenylhydrazone), which depolarizes mitochondria and prevents calcium uptake, eliminated the mitochondrial channel activity, presumably because calcium uptake into mitochondria during nerve stimulation was abrogated. In keeping with the role for this activity in short term plasticity, FCCP application eliminated posttetanic potentiation of the synapse following high frequency nerve stimulation.

## Bcl-xL regulates mitochondrial ATP release during synaptic activity

The above studies raised the possibility that opening of mitochondrial channels produces release of metabolites and/or calcium itself in response to calcium influx. After calcium influx in the synapse, an inner membrane calcium sensitive channel might also be in contact with an outer mitochondrial membrane channel in order to transfer ions and ATP across the outer as well as the inner membrane. The most ubiquitous outer membrane channel is VDAC, which releases metabolites including ATP into the cytosol (Vander Heiden et al., 2001; Gottlieb et al., 2002). In addition to ATP release, VDAC regulates the uptake of ADP and other metabolites during normal and pathological cell activities (Rostovtseva and Colombini, 1997; Mannella and Kinnally, 2008; Maldonado and Lemasters, 2012) and is regulated by cytoskeletal elements, in particular tubulin in its dimeric form (Rostovtseva and Bezrukov, 2012).

The Bcl-2 family also regulates outer membrane channel activity. During programmed cell death, mitochondrial outer membrane permeabilization (MOMP) (Green and Kroemer, 2004; Dejean et al., 2005; Adams and Cory, 2007), is produced by formation of large outer membrane pores comprised of activated oligomerized pro-apoptotic Bax proteins, aided by other pro-apoptotic moieties (Antonsson et al., 2000; Dejean et al., 2005; Kim et al., 2006). In their canonical role, the anti-apoptotic Bcl-2 family proteins such as Bcl-xL protect cells against MOMP by interacting with, and preventing the activities of, the pro-apoptotic family members (Galonek and Hardwick, 2006; Adams and Cory, 2007). Another important function of Bcl-xL during cell death stimuli in cancer cells, however, is to increase the release of ATP through enhanced VDAC opening, to help the cell overcome stress and to decrease the probability of MOMP (Vander Heiden et al., 2001; Gottlieb et al., 2002). In one of the first studies of Bcl-2 family proteins in the synapse, it was found that injection of either Bcl-xL or ATP into the squid presynaptic terminal enhanced synaptic transmitter release to a similar magnitude (Jonas et al., 2003). ATP injection also occluded the effect of Bcl-xL, suggesting that the two agents act via the same mechanism. The channel activity of mitochondria during synaptic transmission was also inhibited by small molecule Bcl-xL inhibitors (Hickman et al., 2008) suggesting that presynaptic plasticity may depend on ATP release from mitochondria regulated by the Bcl-2 family. In addition, these findings raised the intriguing question of whether Bcl-xL regulates not only the release but also the manufacture of ATP during synaptic transmission.

## Mitochondrial metabolic plasticity regulates synaptic properties in hippocampal neurons

Unlike in the high fidelity synapse of the squid where long term synaptic responses are relatively unvaried, in the mammalian hippocampus, long-term changes in synaptic transmission in both directions occur, and these changes may underlie learning and memory formation. In cultured hippocampal neurons, overexpression of Bcl-xL produces an increase in mitochondrial targeting to synaptic sites, and an enhancement in spontaneous and evoked synaptic responses (Li et al., 2008, 2013). Depletion of Bcl-xL produces the opposite findings. Furthermore, Bcl-xL overexpression is correlated with structural alterations in the synapse including enhanced expression of synaptic vesicle numbers, increased synaptic vesicle markers and an increase in postsynaptic markers, consistent with an increase in size and number of synapses. The Bcl-xL-dependent increase in localization of mitochondria to the synapse suggests a link between mitochondria and the marked alterations of synaptic structure found in developing and plastic synapses.

## Bcl-xL increases bioenergetic efficiency by closing an uncoupling leak within the ATP synthase

A link between synaptic alterations and metabolism produced by Bcl-xL was provided by studies on cultured hippocampal neurons overexpressing or depleted of Bcl-xL (Alavian et al., 2011; Chen et al., 2011). Overexpression of Bcl-xL in resting neurons led to a large (almost 100%) increase in cytoplasmic ATP levels. Surprisingly, this was accompanied by a decrease in neuronal oxygen uptake, as measured with oxygen-sensitive electrodes positioned over single neurons, and a decrease in aerobic glycolysis, consistent with the notion that Bcl-xL overexpression increases mitochondrial bioenergetic efficiency. Interestingly, Bcl-xL was found to markedly increase oxygen uptake during activity compared to controls, in keeping with previous findings of an increase in mitochondrial biomass and larger synapses (Li et al., 2008; Berman et al., 2009; Alavian et al., 2011). Despite this activity-dependent increase in oxygen uptake, however, calculations revealed that the fraction of total oxygen uptake used to make ATP during activity is much higher in Bcl-xL expressing neurons than controls, consistent with an increase in bioenergetic efficiency (Alavian et al., 2011; Chen et al., 2011). Bcl-xL depletion reversed the effects on metabolism, decreasing ATP production and increasing oxygen uptake by the resting cells.

How could these alterations in metabolic efficiency take place? As stated above, Bcl-xL increases the conductance of VDAC to release ATP from mitochondria in both cancer cells and squiid synapse. In addition to releasing ATP, however, in hippocampus Bcl-xL was found to interact directly with the ATP synthase to maximize the efficiency of production of ATP. Oxidative phosphorylation is the main source of ATP formation in neurons and requires coupling of electron transport (H+ pumping out of the mitochondrial matrix) to ADP phosphorylation [movement of H+ ions through the F_1_F_0_ ATP synthase (Complex-V)]. By measuring H+ ion movement using the pH sensitive dye ACMA in submitochondrial vesicles (SMVs) enriched in ATP synthase, it was demonstrated that a leak of H+ ions was always present in the SMVs, but that the leak was greatly attenuated by the addition of ATP or ADP in the presence of endogenous Bcl-xL. Using multiple biochemical and imaging approaches, the site of interaction of Bcl-xL within the F_1_F_0_ ATP synthase was localized to the beta subunit of the enzymatic portion (F_1_) of the ATP synthase close to where ATP (ADP) normally binds (Alavian et al., 2011; Chen et al., 2011).

Closure of the leak within the ATP synthase in the presence of Bcl-xL produces mitochondrial plasticity in the form of an increase in metabolic efficiency; Bcl-xL aids actively firing neurons to produce increases neurotransmitter release (Jonas et al., 2003; Li et al., 2008, 2013), consistent with the possibility of a still-to-be-proven connection between the increase in metabolic efficiency and the long-term higher efficacy of synaptic transmission found in Bcl-xL expressing neurons.

In contrast, opening of the Bcl-xL-regulated leak results in metabolic inefficiency. This is confirmed in detail in neurons in which Bcl-xL had been genetically depleted. These neurons display a fluctuating mitochondrial membrane potential and a marked depolarization in the presence of the ATP synthase inhibitor oligomycin, underlining a necessity for Bcl-xL in metabolic efficiency and in interaction with the ATP synthase (Chen et al., 2011). The exact location of the leak, within the ATP synthase complex, is suggested by the next set of findings.

## Permeability transition is the great uncoupler

The largest matrix-calcium-regulated uncoupling mechanism in mitochondrial inner membrane is PT. PT comprises a rapid increase in permeability of the mitochondrial inner membrane to solutes causing severe osmotic disarray. If it is not reversed, PT leads to structural breakdown of the mitochondrial matrix accompanied by outer mitochondrial membrane rupture and cell death. The interaction of this kind of mitochondrial cell death with apoptotic death produced by MOMP has been hotly debated. Although the two types of cell death may be overlapping, it is safe to say that PT is associated with necrotic cell death such as is found in ischemia or injury whereas MOMP may have a more important role in development and genetically predetermined death (Dejean et al., 2006; Bonora et al., 2014). Inter-membrane space pro-apoptotic factors such as cytochrome c and Smac/DIABLO are released during both forms of cell death. In MOMP, outer membrane permeabilization leads to release of these factors, whereas in PT, rupture of the outer membrane after inner membrane swelling leads to release of pro-apoptotic factors into the cytosol (Galluzzi et al., 2009). PT has been extensively studied for its role in ischemic injury in brain, heart and other organs as well as in neurodegenerative conditions (Bernardi, 2013; Bonora et al., 2014). PT is induced by calcium influx into the matrix, ROS, inorganic phosphate and intracellular acidification (Szabo et al., 1992). It is inhibited by ATP/ADP and by Mg^2+^ (Kowaltowski et al., 1998; Crompton, 1999). The pharmacological agent most useful for inhibition of PT is cyclosporine A (CsA) which displaces the PT co-factor, cyclophilin D (CypD) from binding (Szabo and Zoratti, 1991; Giorgio et al., 2009). Recent reports have also confirmed the increased opening of PT by polyphosphates, chains of 10–100 s of repeating phosphates linked by ATP-like high energy bonds (Abramov et al., 2007; Seidlmayer et al., 2012) that have also been linked to causing plasma membrane excitability directly (Holmstrom et al., 2013; Stotz et al., 2014).

In recent years there has been much interest in identifying the molecules that form the mitochondrial PT pore (mPTP) and in understanding its regulation. A critical role for VDAC has been identified but VDAC appears to form the outer membrane passageway for a channel that must span both membranes (Baines et al., 2007). The ANT was put forth as a possible pore-forming molecule. Purified ANT forms active channels when reconstituted into proteoliposomes, and its physical interaction with VDAC, hexokinase, and CypD positioned it to be at the center of a supramolecular complex forming PT (Brustovetsky et al., 2002). However, genetic depletion of ANT does not abolish PT and mitochondria isolated from ANT knock out animals still retain the ability to undergo PT (Javadov et al., 2009). In contrast, mitochondria from CypD null mice are extremely resistant to CsA-inhibited, calcium-induced PT and to certain forms of ischemic cell death (Baines et al., 2005; Nakagawa et al., 2005). Although it is not suspected that CypD, which is soluble and not membrane imbedded, forms the PT pore, it is an important pore regulator of the core complex, by binding to the stator arm of the mitochondrial F1Fo ATP synthase, specifically to OSCP (Giorgio et al., 2009); this suggests that the PT pore may be contained within the ATP synthase complex. In support of this, it has been reported that ATP synthase dimers participate in mPTP formation (Giorgio et al., 2013). This idea is attractive because isolated purified ATP synthasomes (or SMVs) contain all the inner membrane PT regulatory components including the phosphate carrier, ANT and CypD as well as most of the proteins found within the ATP synthase complex (Ko et al., 2003; Chen et al., 2004). In addition, subunit c was found to participate in mPTP function within the complex composed of the ATP synthase, ANT, and PiC (Azarashvili et al., 2014). The authors of this report hypothesized that dephosphorylation of subunit c occurs in the presence of high matrix calcium, leading to opening of mPTP and mitochondrial swelling in a CsA-sensitive fashion. Subunit c of F0F1-ATPase might therefore act as a structural or regulatory component of the mPTP complex in a phosphorylation-dependent manner.

## The c-subunit of the F_1_F_0_ ATP synthase forms the mPTP

Any leak of the ATP synthase complex must be present in the membrane-embedded portion (F_O_). The F_O_ is connected to the enzymatic portion (F_1_) by a central stalk and a peripheral arm called the stator. This arm bends over the catalytic subunit. Protons pumped out of the matrix by the electron transport complexes re-enter the matrix by traveling down their electrochemical gradient through a translocator at the junction between the outer wall of subunit-c and subunit-a of the F_O_. The energy dissipated by H+ translocation creates a conformational change in the alpha and beta subunits resulting in the synthesis of ATP from ADP and Pi (Watt et al., 2010). When viewed from the inter-membrane space, the denuded octameric c-subunit appears as a ring with a central pore-like structure that is normally obscured by the F_1_ stalk components gamma, delta and epsilon (Pogoryelov et al., 2007). The c-subunit ring could therefore form an ion conducting channel that would allow for uncoupling if the stalk partially or completely dissociated from it. Older studies suggested that the c-subunit of F_O_ has pore-forming capability (McGeoch and Guidotti, 1997; Pavlov et al., 2005), and a recent study has found that depletion of all three isoforms of c-subunit by siRNA in cells leads to protection from the onset of PT in response to calcium or oxidant challenge (Bonora et al., 2013). In addition, over-expression of the c-subunit under certain conditions can pre-dispose to PT (Bonora et al., 2013).

**Figure 2:**
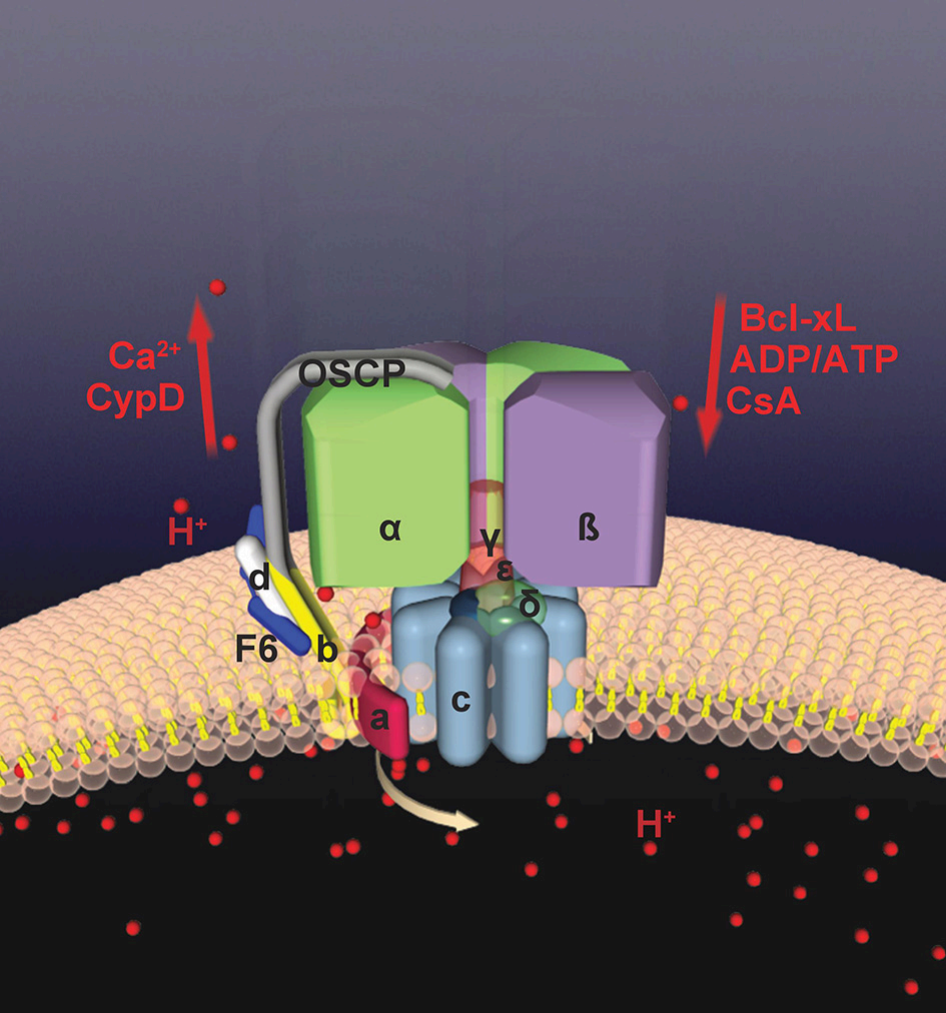
**Bcl-xL binds to ATP synthase and regulates mPTP**. Shown are binding locations between various regulatory molecules and the ATP synthase. The ATP synthase has recently been found to form the mPTP. The c-subunit forms a leak within the ATP synthase which is closed by F1 interaction. Bcl-xL and ATP/ADP close mPTP by interacting with the beta subunit of the F1. CsA and CypD bind to OSCP; CypD enhances opening of mPTP by releasing the F1 from the c-subunit pore, opposed by the actions of CsA.

To test the hypothesis that the c-subunit ring forms the pore of the mPTP, we analyzed the ability of the purified c-subunit to form a pore (Alavian et al., 2014). Indeed, recordings of the purified c-subunit yield a multi-conductance, voltage dependent channel. Reconstructing regulation of the c-subunit pore by recording from increasingly purified groups of inner membrane proteins demonstrate that there are layers of regulation of the c-subunit leak channel. In mitochondria or inner membrane preparations (lacking the outer membrane) calcium activates the c-subunit leak channel while CsA and ATP/ADP inhibit it. Removal of the F_1_ by urea treatment of the inner membrane or removal of CypD by purification of ATP synthase monomers causes the c-subunit channel to lose sensitivity to CsA and calcium but not to ATP/ADP, suggesting that the CsA/calcium binding site is associated with the F_1_ portion but that a second ATP binding site exists in the F_O_. This is consistent with reports identifying the CsA/CypD binding site on OSCP (Giorgio et al., 2009, 2013). Channel activity of the purified c-subunit is inhibited by the purified beta subunit of F_1_ and by ATP/ADP, suggesting a structural rearrangement whereby the stalk of the ATP synthase inhibits opening of the c-subunit leak channel, aided by ADP/ATP binding to the beta subunit or by the pharmacological agent CsA binding to OSCP (Figure 2).

Interestingly, exposure of mitochondria to high calcium unmasks the F_O_ by separating the F_1_ from the F_O_, indicating that calcium activates the PT pore by facilitating this separation. The unmasking of the c-subunit leak channel is prevented by pre-exposing mitochondria to CsA or ADP. In addition, the unmasking of the c-subunit in response to calcium is greatly attenuated in mitochondria isolated from the CypD null mouse (Baines et al., 2005; Nakagawa et al., 2005) that is resistant to calcium induced PT. ATP synthase channel activity is also enhanced by the addition of CypD protein to purified ATP synthase monomers, implicating CypD as a regulator of the c-subunit leak channel, opposed by CsA and ADP (Alavian et al., 2014). Therefore, the leak channel, which has a higher probability of closed than open state in intact and enzymatically active synthase complexes, opens in the presence of CypD and mitochondrial calcium influx by dissociation of the OSCP-bound catalytic subunits. Exposure of the membrane (F_O_) portion and inner mitochondrial membrane depolarization could, therefore, be reversible through an OSCP (or beta subunit)-dependent conformational modification of the complex (Figure 2).

## mPTP opening correlates with cell death in acute ischemia, ROS damage or glutamate excitotoxicity

PT occurs acutely during such events as ischemic excitotoxicity or high ROS production that can occur during rapid onset of brain or cardiac ischemia (Baines, 2009). Our studies and those of others (Bonora et al., 2013) show that excitotoxic and ROS-induced cell death are greatly attenuated upon depletion of the c-subunit by shRNA in neurons and other cells (Bonora et al., 2013; Alavian et al., 2014); however, cell death protection by c-subunit depletion is not further attenuated by CsA, suggesting that the c-subunit forms the inner mitochondrial membrane target of the CsA-sensitive complex (Alavian et al., 2014). Opening of the PT pore under stress may also be sensitive to Bcl-xL binding to the F_1_ beta subunit, as suggested by our recent studies (Chen et al., 2011; Alavian et al., 2014). In contrast to c-subunit depletion, over-expression of the wild type c-subunit (Bonora et al., 2013) or mutation of the c-subunit to form a high-conductance leaky c-subunit (Alavian et al., 2014) predisposes to enhanced cell death upon excitotoxic or ROS stimulation. Death under these conditions is not sensitive to CsA, presumably because the leaky pore prevents normal regulation by components of F1. In summary, our data and other recent studies suggest that the c-subunit of the ATP synthase forms the pore of a large complex of regulatory proteins within the ATP synthase that comprises the mPTP. Upon calcium or ROS exposure, opening of the pore by relative removal of the F_1_ from the membrane produces mitochondrial membrane depolarization, abrogation of the H+ gradient across the inner membrane, and cell death.

## Pro-apoptotic proteolytic cleavage fragment of Bcl-xL causes large conductance mitochondrial ion channel activity correlated with hypoxic synaptic failure

In the synapse, ROS or excitotoxicity may not produce death of the soma, but in contrast severe neuronal stress may result in long lasting synaptic depression or a decline in neuronal excitability. A decline in neurotransmitter release or recycling may subsequently mark a synapse for elimination, followed by somatic death if many synapses are sequentially eliminated. Before the onset of decline, a set of changes occurs in mitochondrial membrane activity that negatively affects synaptic function.

Under pro-apoptotic conditions in growth-factor deprived cancer cell lines, Bcl-2 family proteins activate large mitochondrial outer membrane channel activity that participates in release of pro-apoptotic factors from mitochondria (Antonsson et al., 2000; Dejean et al., 2005), either in the absence of any change to the properties of the inner membrane or, as may occur during ischemia, accompanying induction of calcium or ROS-induced depolarization and loss of osmotic regulation (permeability transition) of the inner mitochondrial membrane (Tornero et al., 2011; D'Orsi et al., 2012; Perez-Pinzon et al., 2012). In the synapse, the effects of hypoxia serve as a model to study the role of Bcl-xL in mitochondrial ion channel events activated during severe neuronal injury (Jonas et al., 2004, 2005). The presynaptic terminal is very sensitive to hypoxia, which attenuates synaptic transmission over 10–30 min. Patch clamp recordings of mitochondrial membranes at rest during hypoxia reveal large conductance activity not found frequently in controls. The channel activity is larger than that induced by pipette-mediated application of recombinant full length Bcl-xL protein and is mimicked by activity of recombinant proteolytically-altered Bcl-xL (ΔN Bcl-xL) that forms a large conductance (pro-apoptotic or MOMP-like) channel activity in the outer mitochondrial membrane. In contrast, large conductance activity of mitochondria during hypoxia is prevented by pre-treatment of the synapse with a pan-caspase/calpain inhibitor that prevents the cleavage of Bcl-xL. Appearance of the channel associated with ΔN Bcl-xL during hypoxia most likely arises from specific proteolysis of Bcl-xL and not from general injury, because levels of VDAC are preserved in both caspase/calpain inhibitor-treated and untreated hypoxic synapses.

In contrast to the response to injection into the synapse of full length Bcl-xL, which causes synaptic potentiation, injection of ΔN Bcl-xL protein produces a marked synaptic depression (Jonas et al., 2003; Hickman et al., 2008). The time course of rundown of synaptic responses matches that of hypoxia, suggesting a correlation between the two types of synaptic decline.

More evidence that Bcl-xL protein can produce two different conductance level channel activities came from *in vivo* studies with the Bcl-xL inhibitor ABT-737. When applied to mitochondria within the squid presynaptic terminal just before healthy synaptic transmission, ABT-737 inhibited the channel activity of mitochondrial membranes induced by synaptic stimulation, suggesting that full length Bcl-xL is necessary for this activity (Hickman et al., 2008). ABT-737, however, also attenuated the channel activity of ΔN Bcl-xL (Hickman et al., 2008). ABT-737 reversed the decline in synaptic responses produced by hypoxia or by direct injection of ΔN Bcl-xL into the terminal and enhanced synaptic function, suggesting that the amplitude of activity at mitochondrial outer membranes may determine the direction of changes in synaptic strength. In addition, by attenuating the channel activity of ΔN Bcl-xL in an *in vivo* model of transient global ischemia, ABT-737 effectively prevented delayed cell death of hippocampal CA1 neurons (Ofengeim et al., 2012). Ischemic death of CA1 neurons was also prevented in a KI mouse containing a form of Bcl-xL resistant to caspase/calpain cleavage (Ofengeim et al., 2012) confirming the specific role of ΔN Bcl-xL in the onset of cell death in hippocampal CA1 neurons during global ischemic brain injury.

## Synaptic responses decline during long term depression in association with Bcl-xL-regulated Bax-induced mitochondrial channel activity

The enhancement of synaptic responses by full length Bcl-xL could be related to a difference not only in size of the conductance of its mitochondrial ion channel compared to that of pro-apoptotic molecules such as Bax or ΔN Bcl-xL, but also to a difference in function of the activity. A key characteristic of the ion channel activity of Bcl-xL is that it can induce ATP exchange across mitochondrial membranes (Vander Heiden et al., 2000, 2001) while activity of ΔN Bcl-xL or Bax causes release of pro-apoptotic factors such as cytochrome c and in addition causes caspase activation. The delicate balance between pro- and anti-apoptotic Bcl-2-related activities may thereby regulate mitochondrial metabolism at times of stress and may control the timing of eventual synaptic rundown or death of the soma if neuronal stress overwhelms anti-apoptotic capabilities (Plas and Thompson, 2002). Release of factors such as cytochrome c not only activates downstream caspases that inactivate cellular processes, but also directly compromises function by depriving mitochondria of electron transport carriers; These features may all contribute to the decline in synaptic responses found after full length Bcl-xL cleavage or Bax activation and formation of large conductance outer membrane channel activity.

## LTD represents normal synaptic plasticity but can be a marker of synaptic degeneration

Long term synaptic depression (LTD) caused by low frequency stimulation or by cell signaling is a normal mechanism of synaptic plasticity opposite in some ways to long term potentiation (LTP) brought on by high frequency stimulation (Malenka and Bear, 2004). Despite its role in normal synaptic plasticity, however, long term depression can also serve as a marker for a pre-degenerative synaptic state. In hippocampal CA3 to CA1 synapse, low synaptic activity leads to a long lasting decline in synaptic efficacy, brought about in part by removal of postsynaptic receptors (Malinow and Malenka, 2002; Kessels and Malinow, 2009); this state can be quite stable and may never lead to somatic demise. It has been described recently that mitochondria are important for a form of LTD associated with normal synaptic plasticity in hippocampal CA1 neurons. In the CA1 dendrite, low frequency activity causes Bcl-xL-sensitive mitochondrially-mediated release of cytochrome c followed by low level activation of caspase 3, which leads to the removal of postsynaptic glutamate receptors from the plasma membrane, resulting in a form of LTD (Li et al., 2010). In addition to these findings, however, degenerative changes may also be associated with LTD. In synapses from Bax^−/−^ mice treated with the toxic Abeta protein, LTD was prevented, implying that Bax actions at mitochondria are necessary for this form of degenerative hippocampal LTD (Olsen and Sheng, 2012; Sheng, 2014). In a model of developmental axonal targeting in spinal neurons, both mitochondrial Bax and caspase 6 activation were found to control axonal loss in response to nerve growth factor withdrawal (Nikolaev et al., 2009). In this scenario, the N-terminus of amyloid precursor protein (APP) bound to death receptor 6 (DR6) to initiate an intracellular cascade resulting in mitochondrial-dependent axonal demise.

The difference between decreased activity and damage was made more clear in a recent study of neurite growth arrest in cultured neurons depleted of Bcl-xL by siRNA. Declining Bcl-xL levels prevented normal outgrowth and branching of neuronal processes over 4 weeks in culture before any somatic death occurred (Park et al., 2014). In contrast to the slowly occurring growth arrest found upon Bcl-xL depletion, loss of neurites takes place with a much more rapid timescale after a brief death stimulus of hypoxia. This hypoxia-induced loss is greatly attenuated by normal endogenous Bcl-xL levels and by depletion of DR6, which prevented neurite disruption especially in cells previously depleted of Bcl-xL. These findings raise the interesting question of whether the upregulation of DR6 and neurite growth arrest in Bcl-xL-depleted neurons are related to metabolic changes in dendritic and axonal mitochondria such as would naturally occur in underused synapses.

In support of this notion, Bcl-xL increases the number of synapses housing mitochondria through its actions on the mitochondrial fission protein Drp1 (Li et al., 2008, 2013; Berman et al., 2009). Synapses containing mitochondria have larger synaptic vesicle clusters by light and electron microscopy, suggesting that mitochondria are crucial for this form of synaptic plasticity. Recent studies suggest that axonal branching may also be regulated by mitochondrial positioning: The MARK and SAD family member kinase NUAK1 helps capture mitochondria at axonal branch points (Courchet et al., 2013; Lewis et al., 2013), possibly related to increased activity at these sites. Mitochondria are known to pause at sites where activity is high and they continue to move past sites where activity is low, regulated by specific docking proteins, kinases, and external growth factor cues (Chada and Hollenbeck, 2003; Saxton and Hollenbeck, 2012; Vaccaro and Kittler, 2013); these processes may contribute to synaptic plasticity (Obashi and Okabe, 2013; Sun et al., 2013). The sites where mitochondria dock in preparation for axon or dendritic branching are sites that may form hot spots for mRNA accumulation, protein translation, and vesicular trafficking, as receptors for neurotransmitters and growth factors are inserted or removed to account for the new functions of growing neuritic processes. These studies emphasize that that cues from the extracellular environment during neuronal development or neuronal plasticity may regulate mitochondrial positioning and metabolism as well as protein translation and vesicular trafficking to determine the eventual outcome of neuronal network formation.

## Conclusions

We have focused on Bcl-xL as an example of a survival protein that has more extensive roles than prevention of cell death. In order to promote neuronal survival, perhaps Bcl-xL responds to neuronal activity by translocating to mitochondrial enhancing the efficiency of metabolism by regulating ATP production synthesis via changes in calcium uptake and release and the efficiency of ATP synthase activity. Thus, under normal circumstances, Bcl-xL coordinates mitochondrial activity with enhanced neurotransmitter vesicle recycling. In the opposite scenario, Bcl-xL protein loss from mitochondria and intracellular organelles may mimic synaptic disuse leading to depression of synaptic responses, pro-apoptotic mitochondrial conductance changes, metabolic compromise and eventual loss of neurite outgrowth. This type of synaptic rundown is also found acutely in hypoxic brain damage, synaptic injury, and in long-term neurodegenerative changes.

At synaptic mitochondria, pro-apoptotic mitochondrial conductance changes regulated by Bcl-xL include not only large conductance activity of the outer mitochondrial membrane, but also changes at the inner membrane characterized by the opening of a large leak conductance found within the ATP synthase c-subunit. We have evidence that this highly regulated leak decreases the efficiency of ATP generation and may lead to acute mitochondrial depolarization and osmotic disarray characteristic of mPT. Bcl-xL, ATP/ADP and certain pharmacological agents deter the opening of this leak, rescuing neurons from necrotic and some forms of apoptotic death. On the other hand, increased probability of leak closure within the c-subunit of ATP synthase may lead to long-term enhancement in the efficacy of synaptic responses and perhaps neuroprotection.

### Conflict of interest statement

The authors declare that the research was conducted in the absence of any commercial or financial relationships that could be construed as a potential conflict of interest.
